# Induction of excitatory brain state governs plastic functional changes in visual cortical topology

**DOI:** 10.1007/s00429-023-02730-y

**Published:** 2023-12-02

**Authors:** Ulf T. Eysel, Dirk Jancke

**Affiliations:** 1 Department of Neurophysiology, Ruhr University Bochum, 44780 Bochum, Germany; 2 Optical Imaging Group, Institut für Neuroinformatik, Ruhr University Bochum, 44780 Bochum, Germany

**Keywords:** Cortical plasticity, Hyperexcitability, Response variability, Visual cortex, Retinal lesion, Functional reorganization, Transcranial magnetic stimulation (TMS)

## Abstract

Adult visual plasticity underlying local remodeling of the cortical circuitry in vivo appears to be associated with a spatiotemporal pattern of strongly increased spontaneous and evoked activity of populations of cells. Here we review and discuss pioneering work by us and others about principles of plasticity in the adult visual cortex, starting with our study which showed that a confined lesion in the cat retina causes increased excitability in the affected region in the primary visual cortex accompanied by fine-tuned restructuring of neuronal function. The underlying remodeling processes was further visualized with voltage-sensitive dye (VSD) imaging that allowed a direct tracking of retinal lesion-induced reorganization across horizontal cortical circuitries. Nowadays, application of noninvasive stimulation methods pursues the idea further of increased cortical excitability along with decreased inhibition as key factors for the induction of adult cortical plasticity. We used high-frequency transcranial magnetic stimulation (TMS), for the first time in combination with VSD optical imaging, and provided evidence that TMS-amplified excitability across large pools of neurons forms the basis for noninvasively targeting reorganization of orientation maps in the visual cortex. Our review has been compiled on the basis of these four own studies, which we discuss in the context of historical developments in the field of visual cortical plasticity and the current state of the literature. Overall, we suggest markers of LTP-like cortical changes at mesoscopic population level as a main driving force for the induction of visual plasticity in the adult. Elevations in excitability that predispose towards cortical plasticity are most likely a common property of all cortical modalities. Thus, interventions that increase cortical excitability are a promising starting point to drive perceptual and potentially motor learning in therapeutic applications.

## Visual cortical plasticity in adulthood

Although the growth and loss of neuronal elements are important common features of postnatal development of the brain the underlying mechanisms are downregulated after the critical periods and the coarse structure and function of the adult brain are considered relatively stable after optimization in early life. However, challenges like use and disuse as well as damage seem to reinstall functional as well as structural mechanisms of neuronal plasticity in the adult. Retinal lesions are a reproducible and well documented model for lesion induced cortical plasticity and have been widely used over the last 40 years to study neuronal reorganization in the adult visual system. Our contributions to the investigation of cortical plasticity in the adult visual cortex focus on the role of cortical excitability in the functional dynamics of reorganization making use of perturbations such as employed in the retinal lesion model in cats and rats (Giannikopoulos and Eysel 2006; Palagina et al. 2009) and transcranial magnetic stimulation (TMS) experiments in cats (Kozyrev et al. 2014, 2018). The following review has been compiled based on these four publications, centered around the widely discussed idea that transformation of the cortex into an excitatory state is crucial for functional reorganization processes. We outline the cortical mechanisms of adult visual plasticity—starting from invasive retinal lesion interventions and single electrode recordings towards noninvasive cortical stimulation procedures (TMS) combined with wide-field optical imaging of high spatiotemporal coverage using voltage-sensitive dye (VSD).

## Map reorganization and increased excitability in the primary visual cortex following retinal lesion

Specific functions of cat visual cortical cells become fully developed 2–3 months postnatal at the end of their individual critical periods (Braastad and Heggelund 1985; Daw et al. 1995; Fregnac and Imbert 1984). However, if retinal lesions impact the afferent cortical inputs in adulthood the resulting visually "blind" lesion projection zone (LPZ) in the visual cortex again becomes a site for substantial neuronal plasticity. With time more and more cells regain visual excitability and the gradual filling-in process is characterized by the emergence of displaced receptive fields (RF). Sufficiently small lesions are completely filled-in in animals (Chino et al. 1992, 1995; Gilbert 1992; Gilbert and Wiesel 1992; Heinen and Skavenski 1991; Kaas et al. 1990) and humans (Gerrits and Timmerman 1969; Zur and Ullman 2003). Earlier experiments had discovered a small extent of topographical reorganization after retinal lesions subcortically with RFs displaced by 5° at 15° eccentricity corresponding to 250 µm in the lateral geniculate nucleus (Eysel et al. 1980, 1981; Eysel 1982). A similar displacement of cortical RFs after paracentral lesions revealed a plasticity spanning distances larger than 2.5 mm (Gilbert 1992; Gilbert and Wiesel 1992; Kaas et al. 1990). In the early phase of reorganization cortical cells displayed enlarged RFs immediately inside of the LPZ (Chino et al. 1992; Gilbert and Wiesel 1992). At termination of the following long-term cortical remodeling the enlarged RFs were reduced in size (Gilbert and Wiesel 1992) and several other RF properties showed improvements towards normality (Chino et al. 1995). The following two sections highlight a strong cortical hyperexcitability associated with the spatiotemporal dynamics during the previously described filling-in process and demonstrate the intracortical spread of excitation in the adult visual cortex, using single cell recordings in the cat and 2D-voltage-sensitive dye (VSD) imaging with high temporal resolution in the rat, respectively.

### Dynamic changes of excitability during cortical map reorganization

**Fig. 1:**
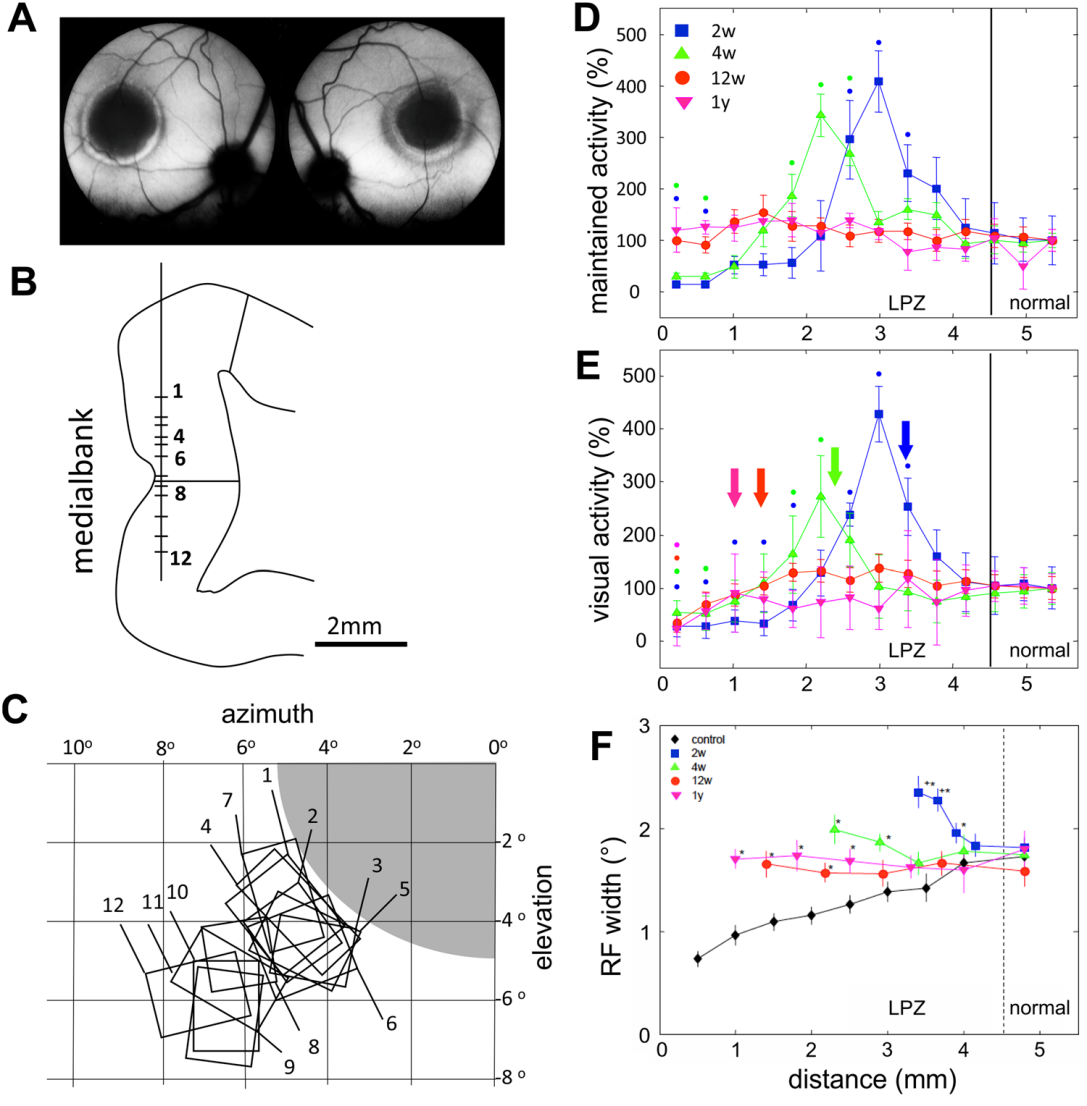
**A** Fundus photography. **B** Vertical recording track down the medial bank in cat area 17. The recording sites of the twelve RFs shown in **C** are indicated. The lines mark the borders between LPZ and normal cortex. **C** Receptive field map corresponding to the recording sites shown in B. The shaded retinal lesion corresponds topographically to the LPZ in **B**. **D** Maintained activity inside and outside of the LPZ in % of the activity in the surrounding normal cortex. Abscissa: Distance of recordings from the cortical surface as depicted in B. Error bars indicate standard errors of the mean. Survival times are color coded. Color coded dots mark significant differences (*p* < 0.05) from normal at given survival time. **E** Strongest responses to visual stimulation with drifting grating with optimal stimulus parameters (orientation, direction). The arrows indicate the first normal RFs at a given survival time. Normalization, color coding, error bars and symbols as in **D**. **F** Receptive field widths in normal controls and in the surround of retinal lesions. Statistically significant differences from normal controls (5% level) are indicated with asterisks. Color code and error bars as in **D**, **E**. For further details see text. (Parts of this figure have originally been published in Giannikopoulos and Eysel 2006, Figs. 1A–C, 3B, C and 5A)

The classical method to demonstrate the reorganization of cortical connectivity following retinal lesions (see Box 1) is the electrophysiological recording of single cells and the determination of their receptive field topography while recording within the LPZ and in the normal cortex (Fig. 1).

**Box 1:** .

**Retinal lesion procedures**	
To study cortical reorganization in cats, a central retinal lesion of approximately 10° in diameter was produced (Fig. 1A) with xenon light (LOG-2 photocoagulator, Clinitex, Inc., USA) in both eyes of adult cats. The optic disks, the vascular pattern, and the retinal lesions (dark pigmented areas) were visible in fundus photographs (Fig. 1A)	
**Electrophysiological recordings in the visual cortex**	
For extracellular recordings an independently controlled linear microelectrode array (305 µm inter-electrode distance) was used. Receptive field topography was first determined with hand-held bar-shaped stimuli for each recording site and plotted as rectangles in the same map (Fig. 1C). Visual responses were then quantified using gratings (0.2–0.7 cycles/deg drifting at 1–2 cycles/s with different orientations (0°–180°) and directions of motion (0°–360°). Recordings were performed in control animals and lesioned animals after survival times of 2 weeks, 1 and 2 months and 1-year

After recovery periods between 2 weeks and 12 months the lesion-induced cortical changes were investigated in acute preparations under continuous anesthesia. Single cortical cells were recorded in the upper layers of cat area 17 moving from inside the LPZ in the upper part of the medial bank downwards into the visual cortex representing the surrounding normal retina (Fig. 1B). In control animals, RFs shifted from the area centralis progressively towards the periphery of the visual field. In lesioned animals, while moving the electrodes downwards through the LPZ the RFs piled up at ectopic positions next to the retinal lesion and only showed a progressive shift when recording further down from the normal cortex.

The maintained activity (Fig. 1D) and the visual responses (for details of recordings see Box 1) were quantitatively determined (Fig. 1E) and depicted as a function of recording location along the electrode track (Fig. 1B).

In control animals, it was found that the relative recording probability of single cells was constant along the vertical recording tracks, but decreased progressively with distance from the normal cortex in lesioned animals and reached about 15% of normal close to the center of the LPZ with recovery times up to 3 months. A significant increase to more than 50% was observed after 1 year of recovery.

Spatiotemporal peaks of significantly increased maintained activity were discovered at 2 and 4 weeks migrating with time towards the center of the LPZ which disappeared upon completion of the filling-in after 12 weeks to 1 year. At this time normal maintained activity was measured throughout the LPZ, while very low maintained activity was observed in the central LPZ (Fig. 1D). At the same recovery times comparable peaks of visual hyperexcitability characterized the newly emerging visual responses inside the LPZ (Fig. 1E). The rising phase and peak of visual hyperexcitability resulted from strong responses to full-field drifting gratings while no well-defined RFs were detected at these recording sites during early reorganization of the LPZ. This spatiotemporal pattern characterized the forefront of visual re-innervation of previously "blind" cells. The well-defined RFs emerged later in time within the LPZ, i.e. closer towards the normally innervated cortex (see arrows in Fig. 1E).

In normal cats, RF size grows larger at larger eccentricities in the visual field as shown along the recording tracks from the cortical surface downwards (Fig. 1F, control). In contrast, at the shorter survival times after lesioning the width of RFs in the LPZ started at significantly larger values and decreased towards the border of the LPZ (Fig. 1F). The widest RFs were detected close to the excitability peak (Fig. 1D, E). At longer survival times (3 and 12 months), the RF widths inside the LPZ recovered and decreased to the sizes found in normal cells close to the LPZ (Fig. 1F).

It can be concluded that increased excitability in general and the increased size of RFs in particular might indicate an imbalance between glutamatergic excitation and GABAergic inhibition in the LPZ of adult cats in early stages of functional reorganization. Two weeks after retinal lesioning the GAD immunoreactivity is downregulated (Arckens et al. 2000; Rosier et al. 1995) and GABA concentrations are decreased. At the same time, a peak of strongly increased Glutamate immunohistochemistry is detected in the LPZ. This peak moves spatiotemporally within about 3 months together with a peak of hyperactivity (Fig. 1D, E) from the border towards the center of the LPZ (Arckens et al. 2000). This is accompanied by a functional filling-in of the scotoma (Giannikopoulos and Eysel 2006). In early postnatal life increased excitation and reduced inhibition are known to promote synaptic plasticity based on the weak GABAergic inhibition and even excitatory effects of GABA (Guo et al. 1997; Lin et al. 1994). Under these conditions, effective long-term potentiation can be elicited (Fox 1995). Electrical stimulation of horizontal fibers elicited LTP in the cat visual cortex (Hirsch and Gilbert 1993), and horizontal axons responded to retinal lesions with axonal sprouting in the adult cat and monkey (Darian-Smith and Gilbert 1994; Yamahachi et al. 2009). On these grounds, we suggest that the hyperexcitability combined with synaptic use in the horizontal cortical fiber system are key mechanisms for synaptic reconnection and functional filling-in following retinal lesions.

In summary, when visual cortical cells are deprived of retinal input by homonymous retinal lesions in adulthood they form an initially unresponsive “blind” LPZ. This cortical region becomes the stage for subsequent neuronal plasticity with visual reactivation and topographic remodeling (Chino et al. 1992, 1995; Gilbert 1992; Gilbert and Wiesel 1992; Heinen and Skavenski 1991; Kaas et al. 1990). Altogether the literature about lesion-induced plasticity suggests that cortical remodeling is accompanied by a temporal loss of inhibition and increase of cortical excitability that appears to promote functional reorganization of the cortical circuitry.

### Strengthening of horizontal cortical connections captured with voltage-sensitive dye imaging

Single cell recordings as described above provide accurate local information about neuronal output. However, because of limits in the density of spatial sampling and the suprathreshold nature of the signals, the underlying analogue synaptic events that drive far-reaching integrative processes cannot be accessed. Moreover, gradual voltage changes in postsynaptic population activity (Jancke 2017, 2018) within the LPZ that accompany recovery processes remain elusive.

Wide-field optical imaging using voltage-sensitive dye (VSD) (see Box 2) permits the depiction of the ongoing state of the cortex through measurements of changes in membrane voltage across millions of neurons under varying input conditions (Onat et al. 2011a). Furthermore, as the emitted fluorescent signals reflect a continuum of membrane potentials, gradual changes in subthreshold activity that spread via long-range cortical connections (Creutzfeldt et al. 1977; Fisken et al. 1975; Gilbert and Wiesel 1979; Rockland and Lund 1982) become unmasked. Consequently, reorganization processes across widely interconnected neurons, including their nonlinear interactions that are not apparent in extracellular spiking, can be tracked over a wide spatial range as postsynaptic activity at mesoscopic population level (Freeman and Barrie 2000; Jancke et al. 1999; Jancke 2000, 2017; Onat et al. 2011a, 2013). Because the optically measured signals provide a direct link to the functional cortical architecture (Arieli et al. 1996; Blasdel and Salama 1986; Bonhoeffer and Grinvald 1991), potential systematic mappings of stimulus parameters and their combinations become accessible in terms of cortical coordinates (Onat et al. 2011b).

**Box 2:** .

**Wide-field optical imaging with voltage-sensitive dyes**	
To capture the time course of reorganization processes along with alterations in the functional characteristics of lateral connectivity at the neuronal population level, wide-field optical imaging in combination with voltage-sensitive dye (VSD) can be a useful tool (Grinvald and Hildesheim 2004; Jancke et al. 2004)	
Using a tandem-lens system (Ratzlaff and Grinvald 1991) and a fast CCD camera, wide-field VSD imaging allows recording of changes in cortical activity across several millimeters of cortex (Berger et al. 2007; Chen et al. 2006; Grinvald et al. 1994; Jancke et al. 2004; Meirovithz et al. 2012; Muller et al. 2014; Onat et al. 2011a, b; Petersen et al. 2003; Roland et al. 2006; Sit et al. 2009) with high temporal (milliseconds) and spatial resolution [50 microns; for a review see Grinvald and Hildesheim (2004)]. In addition, as the method records activity from large pools of neurons with high spatiotemporal resolution, patterns of activity dynamics can be revealed that are currently still not detectable with densely spaced multi-electrode arrays. Moreover, the method avoids biases in sampling of neurons	
Note that due to light scattering, the depth of the tissue staining with dye, and the applicable focal depth, the main source of the fluorescent imaging signals is derived from cortical superficial layers (> 80%) (Grinvald and Hildesheim 2004; Petersen et al. 2003). Thus, any remodeling occurring in deeper cortical layers including thalamo-cortical afferents is unlikely to be accessible. As neurons in layers 5/6 have dendritic arbors up into layers 2/3, deeper cortical layers may nonetheless contribute to the optical signal	

**Fig. 2:**
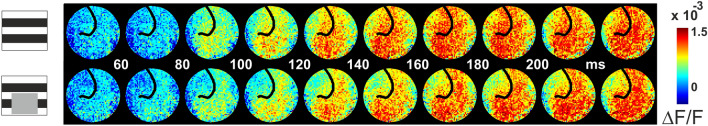
Unmasking horizontal connectivity in rat visual cortex by visualization of propagating activity waves. Top: Stimulation with a “full-field grating (left icon) covering large portions of the visual field. Times after stimulus onset, arrows point to response onset. Bottom: Removal of vertical thalamic input by omitting a part of the grating stimulus, introduces an “artificial scotoma” (see isoluminant gray area in left icon). The border of the scotoma projection zone in the visual cortex is marked by the black line, the upper left region beyond the border does no longer receive vertical input. Instead, note a subsequent “filling-in” of horizontally propagating activity from surrounding regions. Propagation reflects postsynaptic activity that most likely remained subthreshold and is transmitted via horizontal axons (green-yellow colors indicate low amplitude values). (Modified from Supplementary Fig. S1 in Palagina et al. 2009)

One major strength of VSD imaging is its capability to unravel horizontal influence and propagation of activity across upper cortical layers (Grinvald et al. 1994; Jancke et al. 2004; Jancke 2017; Muller et al. 2014; Rekauzke et al. 2016). For a direct proof Fig. 2 demonstrates an example in rat visual cortex, where horizontal propagation was unmasked using an “artificial scotoma” (i.e., hindering retinal feed-forward input using a local isoluminant gray area that omitted a part of the full-field stimulus). Note again that the visualization of such horizontal spread is of particular importance regarding studies of cortical plasticity, as these follow the hypothesis that horizontal connections are a major source of retinal lesion-induced cortical rewiring (Das and Gilbert 1995; Giannikopoulos and Eysel 2006; Kaas et al. 1990).

**Fig. 3:**
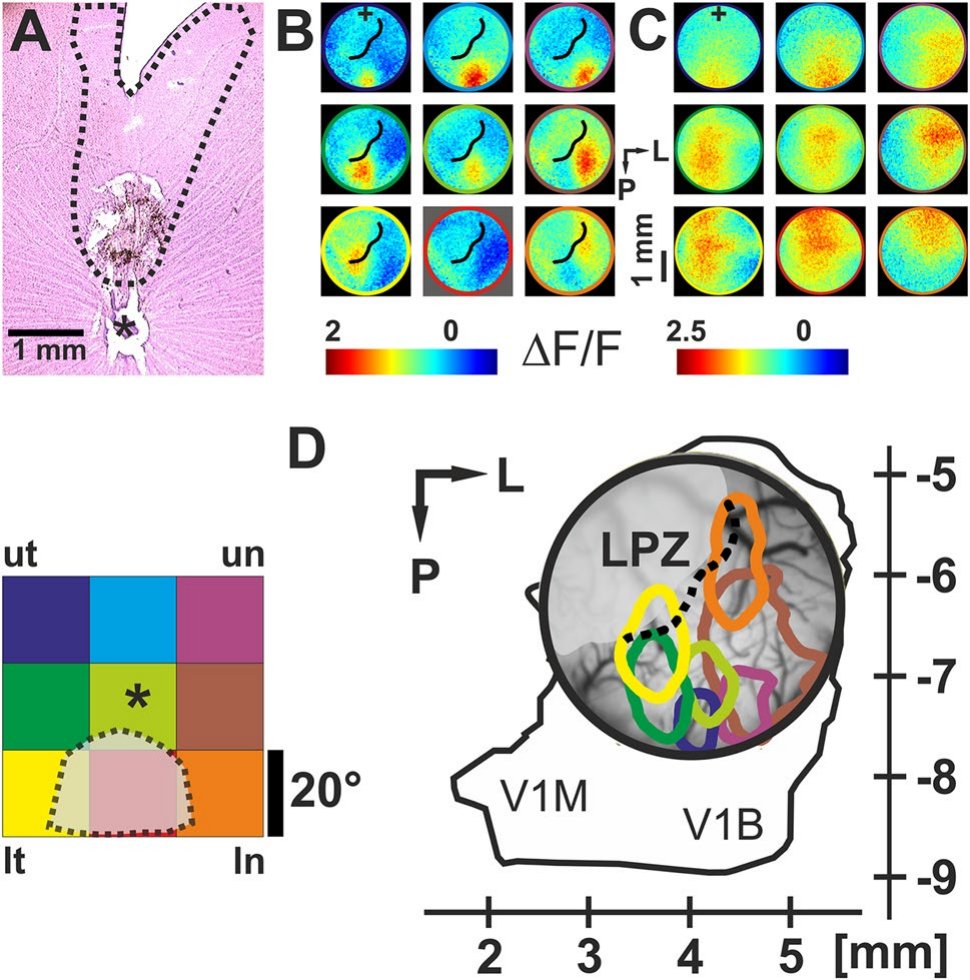
VSD imaging of rat primary visual cortex after local removal of vertical input by a small retinal lesion. **A** Nissl-stained retinal wholemount from left eye. Direct laser lesion of the retina in the dorsal part. Visual loss included also retrograde regions with subsequent ganglion cell degeneration (broken line, asterisk marks optic disc). **B** VSD imaging, 6 days after lesion. Cortical retinotopic representation of 9 visual stimuli (local black/white square-wave grating, individual positions in the visual field are depicted at bottom left, colors are for demonstration purpose only. Asterisk indicates papilla projection). Note that the bottom-middle stimulus (red) did not produce cortical activation (LPZ border is marked by black line). Lesion-affected position in the visual field is shown as gray shaded area (dotted line) at bottom left (ut/lt = upper/lower temporal visual field, un/ln = upper/lower nasal visual field). Colorbar indicates amplitudes of activity (∆*F*/*F*). **C** Same as **B** for an unlesioned animal as a control. Note that the colorbars in B and C have different scales to emphasize the spatial layout of activity in the individual experiments. The lower central stimulus (red) that was unresponsive in the lesioned animal shown in **B** is here represented by evoked activity. **D** Summary plot. Significantly activated regions in **B** are encircled, line colors match stimulus identity, i.e., colors of the nine squares on the left correspond to the circle colors in Panels B and C. Stippled line = LPZ border. Coordinates specify position of the recording chamber relative to Bregma. L = lateral, P = posterior. V1M and V1B = monocular- and binocular part of V1, respectively. Small cross in (B) and (C) correspond to 4 mm L, 5 mm P. (Modified from Palagina et al. 2009)

In our experiments in rats, a laser was used to coagulate a small region (~ 1 mm in diameter) of the retina eliminating all retinal layers dorsal to the optic disc (Fig. 3A). Hence, this intervention produced a retinal lesion that removed feedforward visual input to the medial part of the primary visual cortex, leading to a temporo-nasal scotoma of ~ 15–20° (Fig. 3A, B, D). In this study, following the retinal lesions, VSD imaging was conducted in three groups of animals: the first group, lesioned at P65, was recorded at P69–P72 (“acute lesion”). The second group was also lesioned at P65 but imaged after an extended period of recovery, that is, P92–P105. A third group of unlesioned rats with matching ages was imaged in order to characterize normal retinotopic cortical representations as a control (Fig. 3C).

**Fig. 4:**
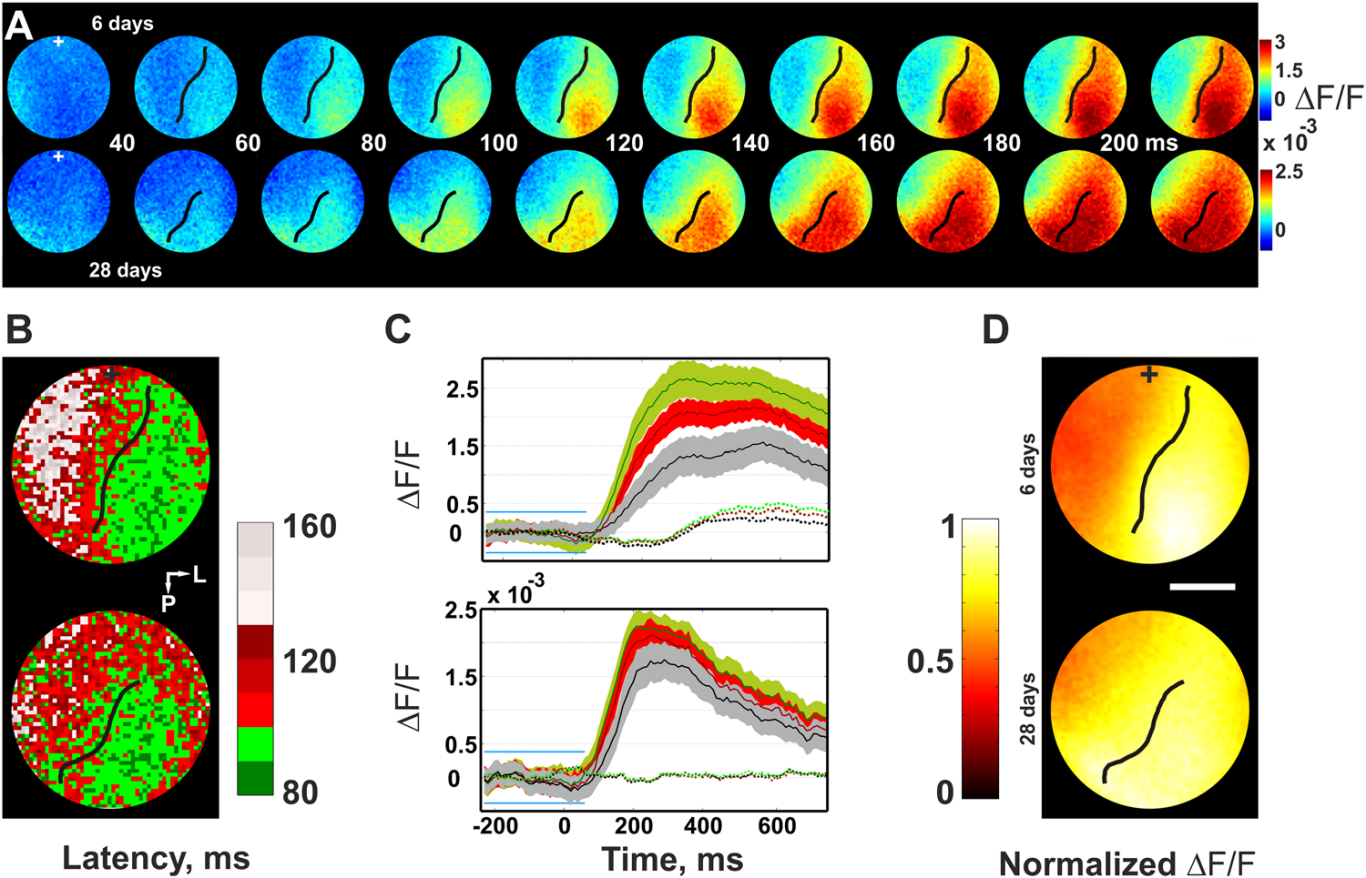
Functional strengthening of horizontal cortical connections after recovery from retinal lesion. **A** Visually evoked activity in rat visual cortex imaged with VSD, same conventions as in Fig. 3B, C. Top: 6 days post-lesion; bottom: 28 days post-lesion. LPZ border is marked by black line. Following 28 days of recovery horizontal spread was reinforced and propagates at increased levels into the lesion projection zone (LPZ). Conversely, after 6 days of recovery horizontal spread stayed at low amplitude levels, cf. colorbars. **B** Latencies across LPZ were reduced after 28 days of recovery (bottom) as compared to 6 days after lesion (top). **C** Time courses of visually evoked activity spatially averaged across 3 different regions: outside LPZ (green), around LPZ border (red), and within LPZ (gray). Top: 6 days after lesion, bottom: > 28 days post-lesion. Traces depict mean over 20 visual stimulus repetitions, colored contours = 1 SD. Dotted lines show baseline activity (± 2 SD, blue lines) without visual stimulation. **D** Normalized amplitudes of activity across lesion-unaffected cortex (right side of LPZ border, black line) and within the LPZ (left side of black line). Top: 6 days after lesion. Values within the LPZ reached ~ 60% of the amplitude values observed for unaffected regions. Bottom: 28 days after lesion. Amplitudes within LPZ increased to ~ 80%. White bars in the images correspond to 1 mm. (Modified from Palagina et al. 2009)

A summary of the cortical retinotopic map obtained after lesion is shown in Fig. 3, where with the exception of the LPZ (stippled line marks LPZ border) that was “cortically blind”, activity in response to all stimuli outside the projection of the retinal lesion was retinotopically arranged in overlapping fashion (compare colors of sketch of stimulus positions in the visual field and contours encircling cortical activation in Fig. 3D, left and right panels, respectively). Recordings of cortical activity in response to stimuli that covered the entire visual field (full-field gratings), including the scotoma, revealed significant differences between groups (Fig. 4), 6 days after lesion (“acute”, upper row) and 28 days post-lesion (“recovery”, lower row). At response onset, cortical regions that were unaffected by the lesion showed a similar instantaneous and broadly distributed emergence of activity in both groups. This reflected most likely maintained early subcortical input to the parts of the cortex outside the LPZ (marked-off by black line). Indeed, the latency maps in Fig. 4B confirmed that the earliest responses were found along the lateral posterior–anterior axis (green colors) where direct thalamic input remained functionally intact. From then on, in both cases (i.e., after short and longer recovery times) latencies increased systematically towards medial regions (red/gray), demonstrating delayed spread of activity crossing the LPZ border via horizontal axons (black line). Thus, lateral input became gradually effective across regions within the LPZ, which can be seen by the systematic shift of the curves in Fig. 4C towards the LPZ [each of the traces depicts spatial averages across unaffected regions (green), around LPZ border (red), and within the LPZ (gray)]. Noteworthy, also the slopes of latency gradients throughout the LPZ became steeper for 28 days post-lesion cases when compared with acutely lesioned animals (Fig. 4C, lower and upper plot, respectively), demonstrating a more rapid succession of activation across the LPZ after longer recovery. Furthermore, amplitudes of activation inside the LPZ reached higher values in animals after long recovery than in acute animals that had only little time for functional retrieval (Fig. 4D).

In summary, this study showed maintained ability of the adult rat visual cortex for plastic reorganization. In particular, VSD imaging enabled in this case the direct visualization of the functional strengthening of lateral connectivity during the time course of recovery from retinal lesion. That is, after retinal lesion (i.e., local removal of direct retinal input), visually evoked activity of neuronal populations inside the LPZ remained initially subthreshold indicated by propagation of low-level activity and further verified through additional extracellular recordings (not shown). Contrastingly, after longer recovery times, the emergence of stimulus-evoked suprathreshold activity within the LPZ was revealed, most likely due to reorganization processes that strengthened horizontal cortical input.

These findings are corroborated and extended by retinal lesion studies by Keck et al. in adult mice, where within 1–2 days the number of inhibitory cell boutons and spines dropped sharply and inhibitory synaptic density and function declined, while the initially depressed excitatory activity returned to normal values due to synaptic scaling. This was followed by a massive restructuring of excitatory connectivity (within 3–63 days), accompanied by stabilization of new connections and functional filling-in of the scotoma (Keck et al. 2008, 2011, 2013); see also review by Sammons and Keck (2015). Altogether, our results in rodents suggest that horizontal connectivity can undergo gradual reinforcement that counteracts lesion-induced loss of visual thalamic projections, similarly as found in our cat experiments (Giannikopoulos and Eysel 2006). Of note, using the retinal lesion model in monkey revealed only little reorganization effects at the border of the cortical LPZ (Smirnakis et al. 2005). These results were obtained with fMRI recordings and may therefore be not sensitive enough to capture post-lesion neuronal organization processes (Calford et al. 2005). On the other hand, it needs to be considered that projections of the length of axons into visual space coordinates are significantly different between rodents and primates. This may facilitate reorganization processes over larger distances in functional space for rodents as compared to primates. Interestingly, Russo et al. (2021) showed recently in humans that focal cortical lesion can result in the deafferentation of connected areas promoting the emergence of sleep-like slow-waves. Thus, besides local state changes in the region around the LPZ, additional long-range effects may contribute to the observed reorganization properties in V1 following retinal lesion.

The crucial point of this review is to present the idea that an induced excitatory cortical state can trigger functional cortical rewiring in the adult. So far, we showed that retinal lesions produce such an excitatory state in the LPZ in the affected cortical region. However, the question arises whether an excitatory cortical state can also be produced locally through noninvasive methods? If this is the case, applications in humans might become feasible that could initiate neuronal rewiring to confined cortical regions.

## Transcranial magnetic stimulation as a tool for the noninvasive facilitation of visual cortical plasticity

Transcranial magnetic stimulation (TMS) is an increasingly applied noninvasive method (see Box 3) for the medical treatment of humans, such as interventions for neurorehabilitation and diagnostics of neurological disorders (Freitas et al. 2013; Hamer et al. 2005; Höflich et al. 1993; Hummel and Cohen 2005; Kleinjung et al. 2005; Lefaucheur et al. 2001; Loo and Mitchell 2005; McKinley et al. 2012). TMS has also been shown to be a valuable instrument in basic brain research. This is firstly because TMS-evoked perturbative effects (Deco et al. 2019) permit area-selective manipulation of cortical function (Jahanshahi and Dirnberger 1999; Pascual-Leone et al. 2000; Walsh and Cowey 2000). In addition, long-lasting alterations in cortical processing can be induced using different TMS protocols in combination with specific contextual (e.g., sensory-motor) protocols that facilitate plasticity and learning (Tegenthoff et al. 2005; Thompson et al. 2008; Waterston and Pack 2010). For instance, by measuring changes in phosphene thresholds repetitive 1 Hz and 10 Hz TMS protocols over the human visual cortex showed opposing plastic effects on excitability (Boroojerdi et al. 2000; Fierro et al. 2005). 10 Hz repetitive TMS across human V1 improved contrast sensitivity of amblyopic ("lazy-eye") patients (Thompson et al. 2008) and modulates performance in visual feature discrimination and detection tasks (Klaes et al. 2003; Romei et al. 2010). Nonetheless, the physiological mechanisms that underlie TMS-induced adaptive perceptual and behavioral changes remain often unclear (Freitas et al. 2013; Gersner et al. 2011; Siebner et al. 2022). One of the major reasons of this gap in knowledge is that neuroimaging "online approaches" (Siebner et al. 2009) that can be used in humans like, EEG (Ilmoniemi et al. 1997), MEG (Kähkönen et al. 2005), fMRI (Bestmann et al. 2008; Bohning et al. 1997; Paus et al. 1997; Roberts et al. 1997) or near-infrared imaging (Parks et al. 2012) are restricted in either spatial or temporal resolutions, or equally limited in both. Alternative recording methods such as the local monitoring of electrical activity, as frequently applied in animal models, are lacking the needed spatial sampling density and demand the use of (invasive) metal fibers (as single electrode or electrode arrays), which in turn generates TMS stimulation artifacts.

**Box 3:** .

**Transcranial magnetic stimulation**	
For TMS a brief strong current is delivered through a coil generating a magnetic field which traverses the scalp (Barker et al. 1985). In turn an electric field is produced that causes current flow leading to excitation within the neuronal tissue under the coil. The method is free of pain because the brain contains no pain receptors. Instead, for example, when applied over the visual cortex TMS pulses can elicit phosphenes, which are local light sensations (Barlow et al. 1947). TMS became a valuable tool in neuroscience as a single TMS pulse produces a focal suppression (“virtual lesion”) within the target area that can be used to infer its relevance for a given task (Walsh and Cowey 1998; Pascual-Leone et al. 2000). High-frequency pulses are often used to measure cortical excitability or to alter the cortical state when applied as pulse trains over longer time periods (Pascual-Leone et al. 1999; Di Lazzaro et al. 2004; Huber et al. 2013). The latter regime was used in our studies	

### Using light to track TMS-induced changes of cortical activity in real-time

One of the major points of this review is to outline the idea that an induced excitatory cortical state, here generated through the use of 10 Hz repetitive transcranial magnetic stimulation (rTMS), may directly be exploitable to target cortical rewiring processes. Insight into the mechanisms underlying such reorganization processes will crucially help creating settings, in which cortical functional loss in humans may partially be restored (or recovery supported) using noninvasive applications that externally stimulate the brain. Next, we therefore describe our experiments that aimed at elucidating cortical reorganization processes after application of TMS followed by subsequent sensory (in our case visual) stimulation during post-TMS excitatory state.

**Fig. 5:**
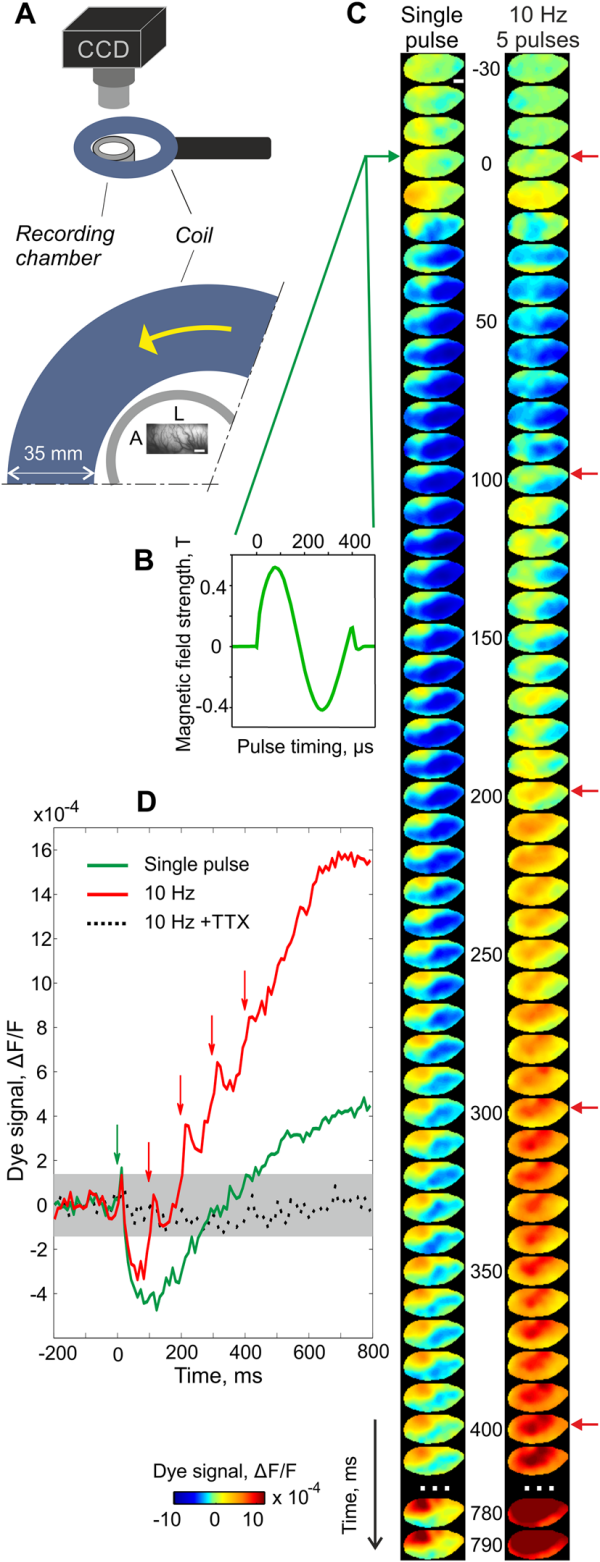
Optical imaging of TMS-induced changes in cortical activity recorded with VSD. **A** Sketch of settings: (gray) recording chamber, (blue) coil and its relative position. Lower plot shows settings from above. Yellow arrow marks the biphasic pulse’s first hemicycle current direction. Image frame depicts vascular pattern across the recorded region (cat V1; L = lateral, A = anterior; bottom white bar = 1 mm). Artefact-free measurements were obtained using a metal-free optical lens and plastic material for the stereotactic platform. **B** Timing and magnetic field strength of applied TMS pulse. **C** TMS-induced cortical dynamics: (left) single TMS pulse (green arrow = onset), (right) 10 Hz TMS (5 pulses, red arrows). The data visualize averages across 40 repetitions expressed as fractional change in fluorescence (∆*F*/*F*, see colorbar at bottom. **D** Time courses of changes in activity (spatial averages of image frames). Gray envelope shows confidence interval (95%) of baseline. Note that in comparison to single pulse TMS, repetitive TMS pulses (10 Hz) produced a stepwise build-up of activity after the early suppression phase present in both conditions. TTX (dotted line) was applied over the cortex to confirm that no electromagnetic TMS artifacts (Conde et al. 2019) were present. (Modified from Kozyrev et al. 2014)

Using fluorescent light to track changes in neuronal activity, VSD imaging avoids signal artifact contamination due to the strong electric field induced by the TMS pulses. Indeed, this optical approach allowed to accomplish, for the first time, artifact-free visualization of TMS-induced activity over several millimeters of cortex in the anesthetized cat with a single image-frame resolution of 10 ms (Fig. 5). Using VSD imaging it could be shown that each TMS pulse (Fig. 5B, left trace) produces a brief focal spot of activation with highest amplitudes evoked close to the coil (cf. Figure 5A, C, first 2 frames after TMS onset, reddish colors). This initial activation was succeeded by strong and widespread suppression lasting up to ~ 300 ms (bluish colors). Thus, a single TMS pulse caused a brief ~ 20 ms period of excitation succeeded by an instantaneous decrease in the postsynaptic potential of a large amount of neurons below baseline (i.e., producing a so-called “vertical lesion”). Following suppression, activity showed a rebound (Moliadze et al. 2003) during which locally rising activity was surrounded by cortical regions that remained suppressed. Altogether, these observations suggest that TMS releases a powerful volley that may initially drive interneurons (Lenz et al. 2016; Murphy et al. 2016; Werhahn et al. 1999; Ziemann 2003), most likely inhibitory neurons expressing parvalbumin (PV) (Benali et al. 2011). As PV neurons target pyramidal neurons on the soma (Markram et al. 2004) and given their far-reaching axonal-dendritic structure (Chung et al. 2013), which may produce enhanced TMS-sensitivity (McAllister et al. 2009), PV interneurons likely impose synchronized inhibition in the cortical circuitries (Pascual-Leone et al. 2000). In turn, excitatory networks could be activated that counterbalance such initially TMS-evoked inhibition, presumably involving activation of NMDA-receptors (Huang et al. 2007).

How are further consecutive TMS pulses represented in the cortex and how do they influence the progression of activity dynamics? After the early phase of suppression following the first TMS pulse, high-frequency TMS (10 Hz) applied over the visual cortex (Aydin-Abidin et al. 2006; Bohotin et al. 2002) induced consecutive boosts in activity in close synchrony with each TMS pulse. Hence, in this case, activity builds-up to high levels with widespread activation (see right trace in Fig. 5C). In Fig. 5D time traces of spatially averaged activity are shown. It can be noticed that in the 10 Hz condition (red trace) the strength of suppression after the first-pulse appears moderately smaller than for the single-pulse protocol (Fig. 5D, compare red and green trace, respectively). We speculate that multiple repetitions of TMS trials lead to enhanced activity that accumulates during high-frequency trials, possibly driven by reduced efficacy of inhibition (Lenz et al. 2016). Overall, repetitive 10 Hz TMS-pulses increased step-wise the level of postsynaptic potentials, starting from initial suppression up to high amplitudes producing an excitatory state across a large pool of cortical neurons (Kozyrev et al. 2014).

### Targeted remodeling of visual cortical maps through TMS treatment

**Fig. 6:**
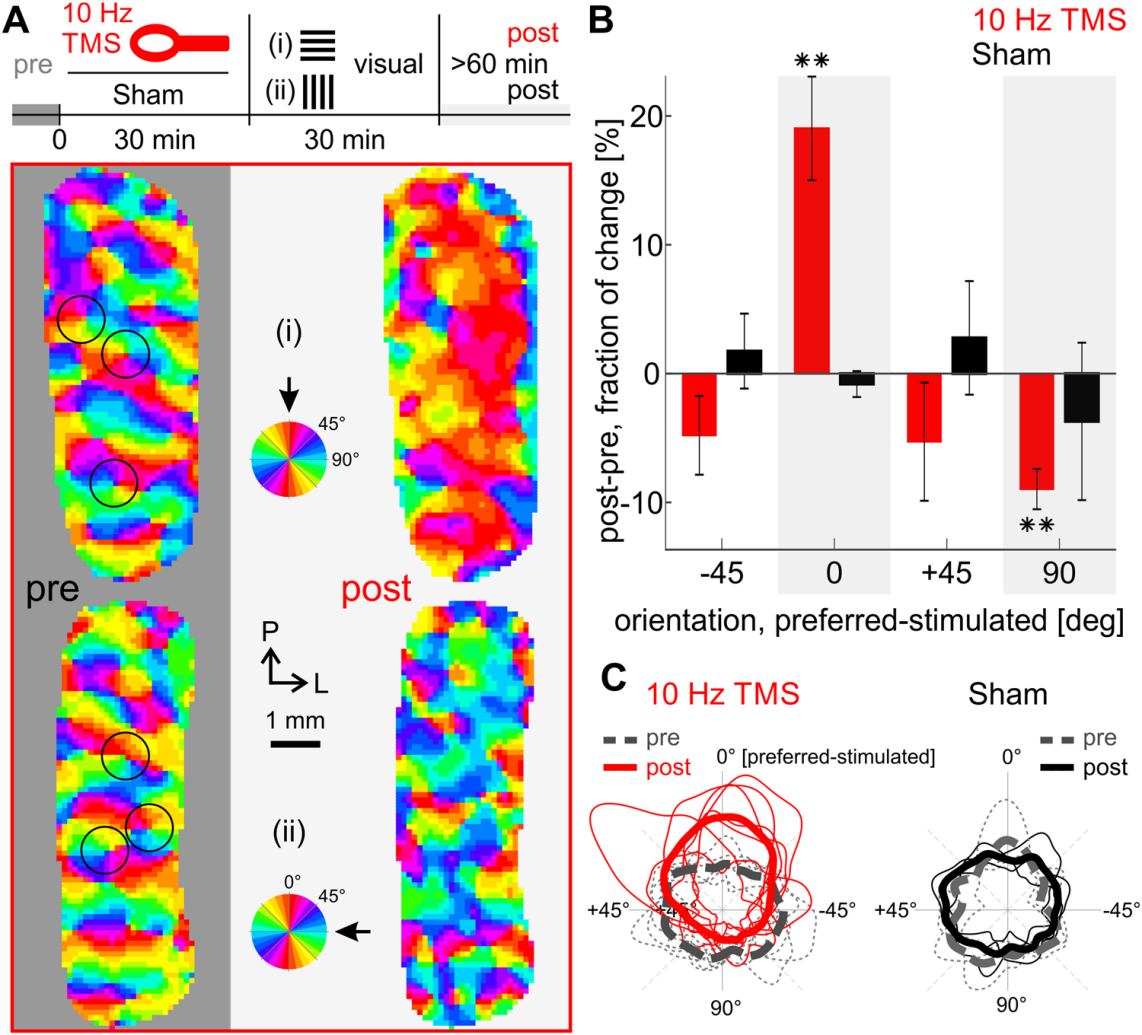
Remodeling the functional layout of adult cat visual cortex through rTMS and visual stimulation. **A** Top panel: Experimental procedure. 10 Hz rTMS (sham TMS as a control) and subsequent visual stimulation; exemplified are 2 orthogonal orientations (i/ii). Bottom panel: Layout of orientation maps, (left) pre-treatment orientation map and (right) after 10 Hz rTMS followed by specific visual stimulation (black arrows indicate orientation used for stimulation). Colors denote the pixels’ orientation preference. Black circles Examples of pinwheel locations (see main text). L, lateral; P, posterior. **B** Changes in preferred orientation. Red: after 10 Hz rTMS and visual stimulation (mean across 7 experiments). Black: sham TMS and visual stimulation (mean across 3 experiments). Error bars show *SEM*. Values were centered relative to stimulated orientation. **p < 0.01, one-sample t test, Bonferroni corrected. **C** Distributions of orientation preference values of individual experiments (thin lines; mean = thick lines), 0° denotes “trained” orientation. (Modified from Kozyrev et al. 2018)

In our experiments in the cat visual cortex, the baseline status of orientation map layout was determined before application of TMS (pre-treatment maps). These maps were then re-evaluated directly after rTMS and after a subsequent “passive visual training” protocol lasting ~ 30 min. The visual stimulation protocol consisted of repeated presentations of gratings (phase-shifted) with a single orientation (Fig. 6A, sketch on top). Two examples of orientation maps before (pre) and after treatment with high-frequency rTMS (10 Hz) and after visual stimulation (post) are shown in Fig. 6A. Pre-TMS maps were characterized by a systematic and balanced representation of orientation angles centered around so-called “pinwheel” centers (Fig. 6A, left column). Thus, the maps before application of the protocol displayed their typical and well-known layout (Bonhoeffer and Grinvald 1991) with roughly equal incidence of all orientations. Next, the maps after 10 Hz rTMS and the following prolonged exposure to a selected orientation (visual “training”) were re-evaluated. The timespan directly after visual stimulation was excluded from analysis to avoid confound with early adaptation effects (Dragoi et al. 2000). We discovered that the maps were now dominated by regions representing the stimulated orientation (see Fig. 6A upper row after “training” with horizontal (0°) orientation). In fact, the cortical area encoding the “trained” orientation, here horizontal (see orange-reddish colors) increased by 28.9%, as measured in a time interval of 1–2.5 h after visual stimulation. This plasticity effect was independent of the orientation used for “training”, as verified by the use of different orientations in the individual experiments. For instance, in the experiment depicted in Fig. 6A bottom row, the stimulus orientation was 90°. In this case, orientation dominance shifted towards vertical (see bluish-greenish colors), revealing an increase (18.6%) in the representation of orientations that again matched closely the stimulated orientation.

The relative enlargement of regions encoding the “trained” orientation was on average 19.0 ± 4.0% *SEM* (7 experiments). Conversely, a significant reduction of the number of pixels with orientation preferences that were orthogonal to the stimulated orientation was observed (see Fig. 6B, red bar at 90°). Small but no significant changes of orientation maps were detected when the visual stimulation was carried out in combination with sham TMS (Fig. 6B, black bars, 3 experiments). Therefore, the reorganization of the cortical maps was evident by an increased occurrence of orientations that represented the visually “trained” and neighboring orientations when 10 Hz rTMS was coupled to subsequent visual stimulation (Fig. 6C, thin red lines in left graph show distribution of orientation preference after 10 Hz TMS for the individual experiments). Additional analysis showed that the TMS- and “training”-induced bias in the remodeled map layouts was not randomly recruited. Instead, remodeling was characterized by a systematic shift of orientation preferences towards the targeted visually “trained” orientation (cf. Fig. 3 in Kozyrev et al. 2018).

**Fig. 7:**
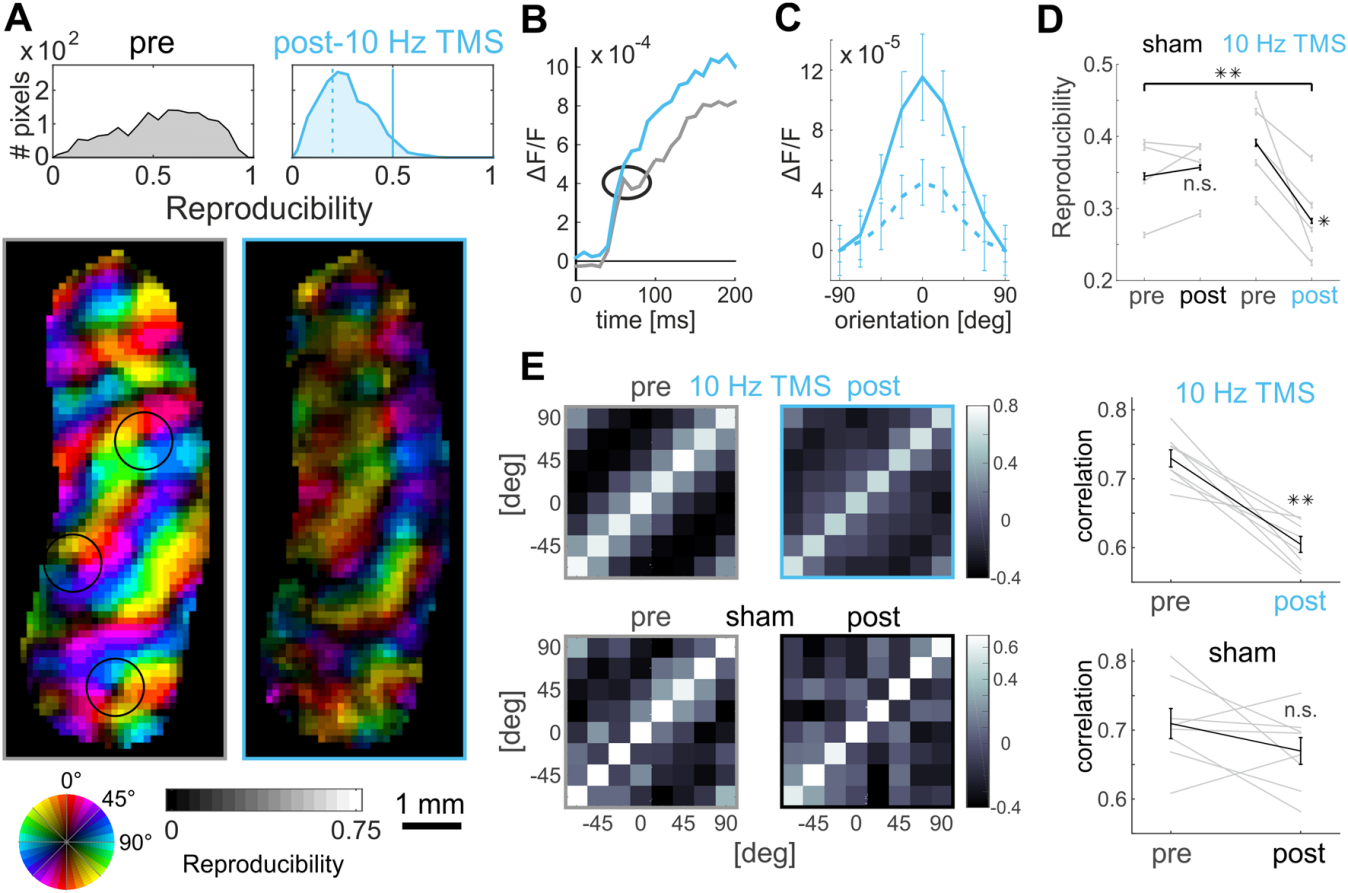
Summary of immediate high frequency rTMS (10 Hz) effects in the visual cortex. **A** Reduced reproducibility. Circle at bottom denotes preferred orientation, hue indicates reproducibility values, right colorbar assigns their range. Distribution of all pixel values across the maps shown in histograms at top. **B** Graphs depict time course of visual responses (spatial averages across the maps shown in **A**). Notch (see circle) present before TMS (grey) was diminished after 10 HZ rTMS (blue). **C** Tuning curves over pixels (mean response amplitude vs. orientation is depicted). Tuning curves of pixels with low reproducibility values, 0–0.2, stippled line (as in **A**), display lower modulation depth (see text) than pixels with high reproducibility values, 0.5–0.7, solid line (as in **A**). Mean of 5 experiments imaged directly after 10 Hz rTMS. **D** Reproducibility values (mean across orientation maps) of individual experiments (grey) and averages (black). **p < 0.01, *p < 0.05, *t* test. **E** Decrease in correlations. Each plot shows a matrix of correlations coefficients (colorbars at right show range of correlation values) between pairs of orientation maps (median of 1000 iterations) averaged over 5 (upper) and 4 experiments (lower). Rightmost graphs summarize individual values of the diagonals of the matrices (grey), averages in black. Error bars show *SEM*. **p < 0.01, Wilcoxon signed rank. (Modified from Kozyrev et al. 2018)

The above observations clearly suggest that the key mechanisms underlying significant remodeling of the functional cortical architecture are generated by means of high-frequency 10 Hz rTMS. Of note, no plastic changes were detected after application of 1 Hz rTMS. Therefore, further analysis of the time window directly after the 10 Hz rTMS treatment (“post-10 Hz TMS”) was performed. The left image in Fig. 7A depicts the layout of an orientation map, this time assigning reproducibility values to all pixels (brightness in maps indicates strength in reproducibility). In brief, if a pixel response was consistently strongest for a certain orientation in each trial (i.e., displaying low single-pixel trial-to-trial circular variance; for in depth explanations of the procedure see Grabska-Barwinska et al. 2009, 2012), a high reproducibility score is assigned. Thus, in Fig. 7A low values (dark) indicate that preferred orientation was inconsistent across trials. Interestingly, and as a proof of concept, this analysis also locates the position of pinwheel centers (cf. dark small regions in the center of encircled areas). Around pinwheel centers (Bonhoeffer and Grinvald 1991) neurons are tuned to different orientations in close vicinity. Hence, their spatial average across pixels leads to low reproducibility values. Using this approach, it could be revealed that the reproducibility of orientation preference for each image pixel was strongly declined after 10 Hz rTMS as compared to the map obtained before TMS (Fig. 7A, compare right (post) and left frame (pre), respectively).

One major cause of increased response variability after 10 Hz rTMS could be reduced inhibition. Note that before application of rTMS, the response to visual stimulation displayed a “notch”, i.e., a small downturn during the early rising phase of activity (Fig. 7B, gray time course). Such notch can typically be found in visually evoked VSD signals and is considered as a signature of inhibition, which may sharpen neuronal tuning to orientation (Sharon and Grinvald 2002). We found that after 10 Hz rTMS the notch was attenuated and activity continued rising towards higher amplitudes as compared to pre-TMS (Fig. 7, see encircled region and further progression of time courses, bluish and gray traces, respectively). Taken together, these results indeed suggest that following 10 Hz rTMS inhibition is reduced, which may facilitate an excitatory cortical state, where amplitudes of sensory-evoked activity are increased and orientation-specific response constituents are weakened (Kozyrev et al. 2014). Consequently, orientation selectivity, calculated as the difference between preferred and orthogonal responses (i.e., “modulation depth”), was significantly reduced for pixels with low as compared to pixels with high reproducibility (Fig. 7C; for a similar observation using intrinsic optical imaging and electrophysiology see (Kim et al. 2015). Moreover, the rTMS-induced loss in orientation selectivity manifested in decorrelated activity across the neuronal circuitries. Correlating pre-and post-TMS orientation maps in response to various stimulus orientations revealed that correlations after 10 Hz rTMS strongly declined (Fig. 7E, left and right plots in upper row). In summary, our 10 Hz rTMS experiments in cats suggested that high-frequency TMS stimulation produces a brain state that sets the ground for subsequent remodeling of functional cortical connectivity. In the visual cortex, such state was characterized by decreased orientation selectivity, increased excitability along with signatures of reduced inhibition, and decorrelation of neuronal responses.

How sustained were perturbation effects and the subsequent cortical remodeling after the solitary TMS intervention? Because of dye bleaching reliable VSD imaging could be performed maximally ~ 6 h after application of TMS (median data acquisition time was 2.5 h post TMS). Within this time window the observed reorganization of cortical orientation maps stayed stable. However, it appears reasonable to assume that a long-lasting establishment of rTMS-induced functional cortical remodeling requires repeated sessions (Cirillo et al. 2017; Gersner et al. 2011). One possible explanation for the need of multiple TMS sessions might be that adult neuronal networks need to overcome “plasticity thresholds” to prevent reestablishment of their pre-treatment properties (Rose et al. 2016). It also should be considered that the described experiments were performed under anesthesia, which can critically change neuronal responsiveness (Niell and Stryker 2010). Thus, results could deviate when subjects receive repeated TMS interventions in the awake state. Nonetheless, several findings speak to the fact that remodeling of cortical maps depends also on structural plastic changes. For instance, de novo formation of axonal boutons along with spine growth and their retraction occur over only tens of minutes (Holtmaat and Svoboda 2009). Moreover, in retinal lesion experiments it was demonstrated that such intensified dendritic synaptic spine turnover could contribute to restructuring of cortical circuitries on a relatively large spatial scale (Keck et al. 2008).

Finally, it remains to be clarified whether TMS-induced cortical remodeling includes additional plastic changes in cortico-thalamic connections (Jaepel et al. 2017) and to which extent remodeling may uncover existing intrinsic connectivity by a potential unmasking of latent inhibitory connections (Barron et al. 2017; Calford et al. 1999; Gilbert and Wiesel 1992). High-frequency TMS is well-known to facilitate cortical disinhibition (Cash et al. 2010, 2016) which in turn likely causes increased excitability as seen in our experiments. In entorhinohippocampal slices 10 Hz magnetic stimulation was shown to decrease the strength of GABAergic synaptic input (Lenz et al. 2016). Thus, the 10 Hz rTMS-induced shift in orientation preference observed in our studies towards the “trained” orientation could emerge from functional weakening of lateral suppression (Patterson et al. 2013). Furthermore, based on the observation that cortical activity displays a strong rebound and excitation phase after prolonged TMS application (Kozyrev et al. 2014; Li et al. 2017), it was recently suggested that the rTMS-induced increase in excitability also involves strengthening of cortico-subcortical loops (Li et al. 2017). Thus, we propose that cortical remodeling by rTMS initially triggers increased cortical excitability in parallel with a cascade of changes in functional strength of connectivity including cortico-thalamic input.

## Concluding remarks and future directions

Overall, in our review we summarized how plasticity in the primary visual cortex is supported by increased excitability, most conceivably triggered through changes in excitation-inhibition balance—a crucial driving force for plasticity in neuronal function (Antal et al. 2006, 2011; Bavelier et al. 2010; Isaacson and Scanziani 2011; Letzkus et al. 2015). Our review adds to the notion that the adult visual intra-cortical network is principally capable of undergoing plastic changes in the basic layout of its functional properties. Specifically, we support the view that adult visual cortical circuitries have the *intrinsic* potential to create functional re-mapping (Calford et al. 1999; Darian-Smith and Gilbert 1994; Das and Gilbert 1995; Giannikopoulos and Eysel 2006; Gilbert and Wiesel 1992; Godde et al. 2002; Kaas et al. 1990; Palagina et al. 2009).

Using small retinal lesions in order to locally and functionally deprive feed-forward input, our studies in adult cats and rats revealed a cortical region of increased excitability, i.e., subsequent hyperactivity, within the lesion-corresponding cortical area followed by substantial reorganization (Giannikopoulos and Eysel 2006; Palagina et al. 2009). We further suggest that visual cortical re-mapping can be accomplished noninvasively with TMS stimulation and specifically targeted to defined plastic changes when combined with selective visual “training” (Kozyrev et al. 2014, 2018). We demonstrate that high frequency rTMS (10 Hz) creates a cortical state where responses to sensory input are “destabilized” apparent by increased variability of evoked activity. In such state, the cortex may transiently undergo a high sensitivity to biases in sensory input (Brickwedde et al. 2019; Clopath et al. 2017; Froemke et al. 2007; Schuett et al. 2001; Sczesny-Kaiser et al. 2014, 2016)—here exercised by visual stimulation with a specific orientation over a longer time period (Bharmauria et al. 2022).

**Fig. 8:**
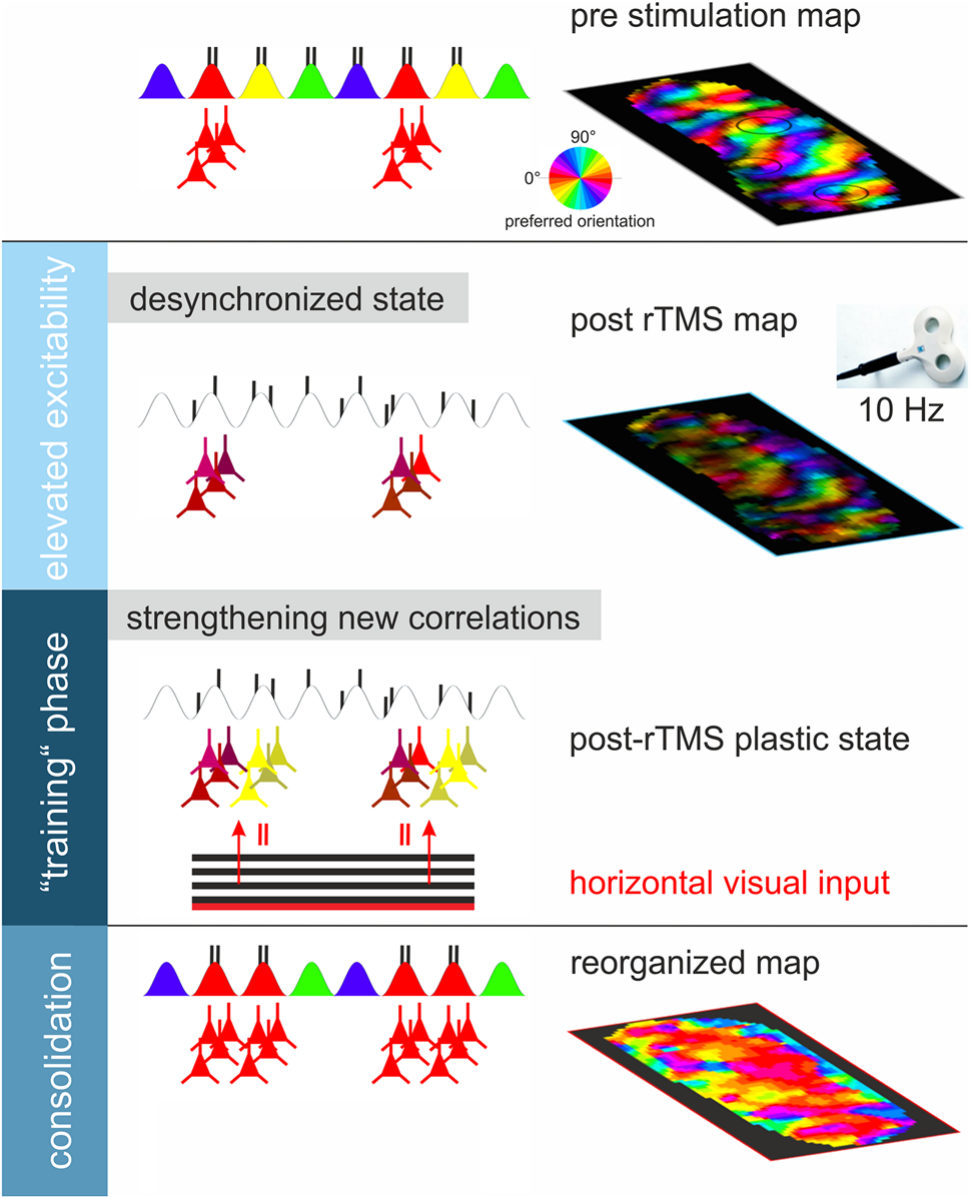
Summary sketch. Top: Topologic layout of orientation maps in the early visual cortex. Neurons coding for different orientations (visualized by different colors) are systematically arranged within orientation maps (pre stimulation map). Neurons encoding a certain orientation (examples tuned to horizontal (0°) are depicted in red) produce highly correlated activity (small vertical lines sketch single spikes) in response to their preferred stimulus orientation. 2nd row: Non-invasive interventions such as TMS at high frequency are capable to induce a cortical desynchronized state of elevated excitability characterized by increased response variability (visualized by dark hue in the map shown at right). 3rd row: This cortical state is sensitive for plastic reorganization processes based on new correlative firing (sketched by red vertical bars), which can be exploited by application of systematic input (in this case by prolonged stimulation with a horizontally oriented grating) that induces and strengthens newly acquired synaptic properties (see yellow neurons originally tuned to oblique orientation). Bottom: Eventually such intervention leads to stable changes of the topologic layout of the cortical circuitries, in which the preference of encoding has been altered dependent on the “trained input”

We show that immediately after high frequency rTMS, the cortex is primed to relearn new connectivity patterns (see summary sketch in Fig. 8). Specifically, increased variability in cortical responses found after high frequency rTMS (Goldsworthy et al. 2021; Kozyrev et al. 2018; Pasley et al. 2009) may facilitate sensitivity to new sensory input regularities, as synapses are exposed to a correlational state above levels reached during normal sensory processing (Fregnac et al. 2010). Indeed, also in the motor domain recent studies revealed promising learning effects after noninvasive brain stimulation protocols (Reis et al. 2009; Suppa et al. 2017; Wessel et al. 2021; Zimerman et al. 2013), in which motor response variability (Wu et al. 2014) may effectively drive motor learning abilities (Teo et al. 2011) and may help to guide therapeutic interventions (Goldsworthy et al. 2021).

## Data Availability

Enquiries about data availability should be directed to the authors.
